# Zonisamide Ameliorates Cognitive Impairment by Inhibiting ER Stress in a Mouse Model of Type 2 Diabetes Mellitus

**DOI:** 10.3389/fnagi.2020.00192

**Published:** 2020-07-09

**Authors:** Yong-Xiang He, Qi-Ying Shen, Jia-Hui Tian, Qian Wu, Qin Xue, Gui-Ping Zhang, Wei Wei, Ying-Hua Liu

**Affiliations:** ^1^Department of Pharmacology, Key Laboratory of Molecular Target & Clinical Pharmacology, School of Pharmaceutical Sciences & the Fifth Affiliated Hospital, Guangzhou Medical University, Guangzhou, China; ^2^Department of Pathophysiology, School of Medicine, Institute of Brain Research, Key Laboratory of State Administration of Traditional Chinese Medicine of China, Jinan University, Guangzhou, China

**Keywords:** Zonisamide, type 2 diabetes mellitus, cognitive impairment, ER stress, Tau, Aβ

## Abstract

Type 2 diabetes mellitus (T2DM) increases the risk of Alzheimer’s disease (AD)-like dementia and pathology. Endoplasmic reticulum stress (ERS) plays a key role in the development of cognitive impairment in T2DM. Zonisamide (ZNS) was found to suppress ERS-induced neuronal cell damage in the experimental models of Parkinson’s disease (PD). However, the protective effect of Zonisamide in the treatment of diabetes-related dementia is not determined. Here, we studied whether ZNS can attenuate cognitive impairments in T2DM mice. C57BL/6J mice were fed with a high-fat diet (HFD) and received one intraperitoneal injection of streptozotocin (STZ) to develop T2DM. After the 9-week diet, the mice were orally gavaged with ZNS or vehicle for 16 consecutive weeks. We found that ZNS improved spatial learning and memory ability and slightly attenuated hyperglycemia. In addition, the expression levels of synaptic-related proteins, such as postsynaptic density 95 (PSD95) and synaptophysin, were increased along with the activation of the cyclic AMP response element-binding (CREB) protein and cAMP-dependent protein kinase (PKA) both in the hippocampus and cortex of T2DM mice. Meanwhile, ZNS attenuated Aβ deposition, Tau hyperphosphorylation at Ser-396/404, and also decreased the activity of Tau upstream kinases including extracellular signal-regulated kinase (ERK) and c-Jun N-terminal kinase (JNK). Moreover, ZNS also decreased the ERS hallmark protein levels. These data suggest that ZNS can efficiently prevent cognitive impairment and improve AD-like pathologies by attenuating ERS in T2DM mice.

## Introduction

Type 2 diabetes mellitus (T2DM) is a peripheral disease exhibiting hyperglycemia induced by insulin resistance. Epidemiological, clinical, and pathological studies have shown a causative association between T2DM and cognitive impairment (Bitel et al., 2012; Vagelatos and Eslick, 2013; Feinkohl et al., 2015). The molecular mechanisms between T2DM and Alzheimer’s disease (AD) are complex (Kandimalla et al., 2017), including insulin resistance, advanced glycation end products (AGE), oxidative stress, inflammation, and mitochondrial dysfunction. All these dysfunctions may contribute to AD-like alteration in T2DM such as impaired synaptic plasticity, the formation of neurofibrillary tangles, and cognitive decline (Bitel et al., 2012; Pugazhenthi et al., 2017). Furthermore, accumulating evidence indicates that endoplasmic reticulum (ER) stress plays an important role in the pathogenesis of diabetic encephalopathy (Zhang et al., 2013; Zhao et al., 2015; Kong et al., 2018). The endoplasmic reticulum (ER) is the primary site of protein synthesis, maturation, folding, and transport. ER stress can be activated by high calcium levels, hypoxia, accumulation of misfolded proteins, and glucose and energy deprivation. ER membrane sensors activate three signaling axes [Lai et al., 2007; Hetz et al., 2013; activating transcription factor-6 (ATF6α), IRE-1α/XBP-1s, and PERK/eIF2α-P] to instigate transcription and translational alterations as well as ER-associated degradation (ERAD) to remove misfolded proteins, aiming at restoring cell homeostasis. Unfolded protein response (UPR) is defined as a collection of signaling pathways that respond to ER stress due to the accumulation of misfolded proteins and/or impaired homeostasis. However, under conditions of prolonged and strong ER stress, the adaptive responses mediated by UPR are not sufficient to restore normal cellular function; cells may also continue toward apoptosis under prolonged ER stress. Subsequently, ER stress induced neuronal apoptosis, synaptic plasticity inhibition, and cognitive impairment (Chen et al., 2003; Duran-Aniotz et al., 2014; Sen et al., 2017; Lin et al., 2018).

Zonisamide (1,2-benzisoxazole-3-methanesulfonate-mide) was developed as an antiepileptic drug (Seino, 2004) as well as approved for use as one of adjunctive therapy for Parkinson’s disease (PD) patients. Zonisamide is known to act through several mechanisms (Holder and Wilfong, 2011), such as modulation of voltage-gated ion channels, increasing extracellular γ-aminobutyric acid (GABA) and the levels of manganese superoxide dismutase, and inhibiting caspase-3 activation. Besides, Zonisamide was found inhibiting ER stress and neuronal cell death through upregulating HMG-CoA reductase degradation 1 (Hrd1) protein levels in the neuronal cell and PD patients (Omura et al., 2013). Endoplasmic reticulum stress (ERS) was known to induce cognitive decline in T2DM mice (Zhang et al., 2013; Zhao et al., 2015; Kong et al., 2018). Thus, Zonisamide may exert neuroprotective actions by regulating ER stress, independent of its antiepileptic activity.

Consequently, we hypothesized that Zonisamide has a protective effect on AD-like alterations by regulating ER stress. In this study, we have evaluated this hypothesis in the HFD-streptozotocin (STZ)-induced T2DM mice.

## Materials and Methods

### Reagents and Antibodies

ZNS was purchased from Target Molecule Corporation (Boston, MA, USA). STZ was purchased from Sigma Chemicals (St. Louis, MO, USA). The main antibodies used in this study are listed in Table 1. Peroxidase-conjugated antirabbit and antimouse immunoglobulin G (IgG) were obtained from Cell Signaling Technology (Danvers, MA, USA). The enhanced chemiluminescence (ECL) kit was obtained from Thermo Scientific (Rockford, IL, USA).

**Table 1:** Primary antibodies used in this study.

Antibody	Type	Specificity	Dilution for WB	Source/reference	Cat. No.	RRID
GAPDH	mAb	GAPDH	1:1,000	ZSGB-BIO	TA08	AB_2747414
Tubulin	mAb	β-Tubulin	1:1,000	Cell Signaling Technology (CST)	2128	AB_823664
PKA-Cα	pAb	α subunit of catalytic subunit in PKA	1:1,000	CST	4782	AB_2170170
PKA-RI	pAb	α/β subunit of Regulatory subunit in PKA	1:1,000	CST	3927	AB_1658217
CREB	mAb	Total CREB	1:1,000	CST	9197	AB_331277
p-CREB	pAb	p-CREB at Ser133	1:1,000	Affinity	AF3189	AB_2156614
PSD95	mAb	PSD5	1:1,000	CST	3450	AB_2292883
Synaptophysin	mAb	Synaptophysin	1:1,000	CST	5461	AB_10698743
Aβ	mAb	β-Amyloid (1–42 prefered)	1:100	CST	14974	AB_2798671
PHF-1	mAb	p-Tau at Ser396/404	1:500	(Greenberg et al., 1992)	Gifted by Dr. Peter Davies	AB_2315150
Tau-5	mAb	Tau	1:1,000	Abcam	ab80579	AB_1603723
JNK	pAb	JNK1, JNK2 or JNK3	1:1,000	CST	9252	AB_2250373
p-JNK	pAb	p-JNK at Thr183/Tyr185	1:1,000	CST	9251	AB_331659
ERK	mAb	Erk1/Erk2	1:1,000	CST	4695	AB_390779
p-ERK	mAb	p-Erk1/p-Erk2 Thr202/Tyr204	1:1,000	CST	4370S	AB_2315112
ATF6	pAb	ATF6	1:1,000	CST	65880	AB_2799696
PERK	mAb	PERK	1:1,000	CST	3192	AB_2095847
PERK pThr982	pAb	p-PERK at Thr982	1:1,000	Affinity	DF7576	AB_2833024
ATF4	pAb	ATF4	1:1,000	Affinity	DF6008	AB_2833025
XBP-1s	mAb	XBP-1s	1:1,000	CST	83418	AB_2800016
GRP78	pAb	GRP78	1:1,000	Affinity	AF0729	AB_2833026
Hrd1	mAb	Hrd1	1:1,000	Abcam	ab170901	AB_2833019

### Animals and Experimental Protocols

Four-week-old male C57BL/6J mice were obtained from Guangdong Medical Laboratory Animal Center (Guangzhou, China). The mice were housed with a standard laboratory diet with food and water available, living in a controlled room temperature (22 ± 1°C), humidity (65 ± 5%) with a 12-h light–dark cycle. All animal experiments were carried out in accordance with the National Institute of Health Guide for the Care and Use of Laboratory Animals (NIH publication 8023, revised 1996) and with the approval of the Animal Ethics Welfare Committee of Guangzhou Medical University. After a 3-week high-fat diet (HFD) feeding (D12492, 60 kcal% fat, Guangzhou, China), the diabetic mice were induced insulin resistance, and the diabetic mice intraperitoneally received 85 mg/kg STZ freshly dissolved in citrate buffer (pH 4.5) twice within 72 h. Six hours after the STZ injection, only the mice with hyperglycemia (fasting blood glucose levels >11.6 mmol/l) were considered as the diabetic mice and were selected for studies.

**Figure 1:**
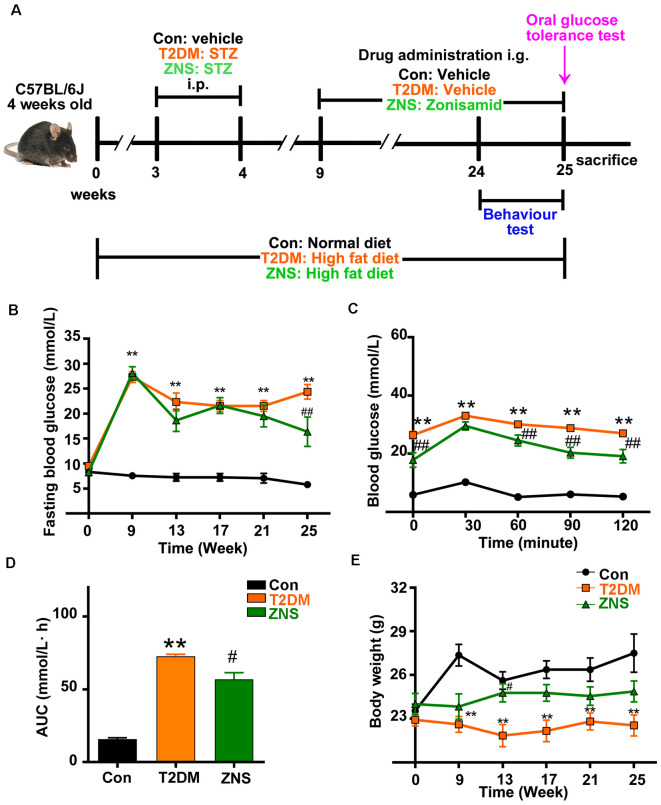
Effects of Zonisamide on body weight and blood glucose of high-fat diet (HFD)/streptozotocin (STZ)-induced type 2 diabetic mice. **(A)** Experimental design scheme for HFD/STZ-induced type 2 diabetic mice. Male 4-week-old C57BL/6J mice were fed either the normal diet (ND) or an HFD for 3 weeks. The HFD-fed mice were intraperitoneally injected (i.p.) with freshly prepared STZ (85 mg/kg) twice within 72 h followed by continued HFD feeding for an additional 5 weeks. From the 9th week, the HFD-fed/STZ-treated mice were orally gavaged (i.g.) with Zonisamide at 40 mg/kg body weight/day or vehicle for 16 weeks. Control mice were on a ND and received vehicle administration. **(B)** Fasting blood glucose levels. **(C)** Blood glucose levels by oral glucose tolerance test (OGTT) after 16 weeks of Zonisamide treatment and **(D)** its area under the curve (AUC). **(E)** Body weight. Data are presented as the mean ± SEM (*n* = 7 mice). **P < 0.01 vs. Con; ^#^*P* < 0.05, ^##^*P* < 0.01 vs. type 2 diabetes mellitus (T2DM).

Mice were divided into three groups; normal diet (ND) mice were taken as controls (Con, *n* = 10), and diabetic mice with HFD were randomly divided into other groups: T2DM (*n* = 10) and the remaining for treatment of Zonisamide (ZNS, *n* = 10). The drug Zonisamide was suspended in 1% dimethyl sulfoxide (DMSO), mice in ZNS group were intragastrically administered Zonisamide (40 mg/kg) daily for 16 weeks, while mice in Con and T2DM groups were given the same volume of 1% DMSO intragastrically. Throughout the whole experiment, the control mice were given a ND, while the diabetic mice were given the HFD. Blood glucose was determined once every 2 weeks (Figure 1A).

All procedures involving animal experimentation were performed in accordance with the recommendations in the Guide for the Care and Use of Laboratory Animals of the National Institutes of Health (USA). The animal use protocol was approved by the Committee on the Ethics of Animal Experiments of Guangzhou Medical University, and the approval reference number was SYXK 2016-0168.

### Blood Glucose Measurement

Mice were fasted for 6 h (morning fasting) before blood glucose measurements. Blood glucose was measured through tail vein bleeding with the use of a glucometer (SD Biosensor, Inc., Korea).

### Oral Glucose Tolerance Test

Briefly, after 16 weeks of Zonisamide treatment, all mice were transferred into the cage with fresh bedding and were fasted 12 h before testing while ensuring that the mice have access to drinking water. Then, glucose solution (2 g/kg of body weight) was administered by oral gavage for every mouse. Blood glucose samples were taken at 0, 30, 60, 90, and 120 min through the caudal vessels and then measured using a glucometer (SD Biosensor, Inc., Korea). The total glycemic response was calculated from different areas under the curve within 120 min of observation period.

### Morris Water Maze

Morris water maze (MWM) was used to assess spatial and memory learning in mice. The Morris labyrinth (120 cm in diameter, 35 cm high) was a white circular circle with no features on the inner surface. The round pool was filled with white dyed water and kept at room temperature (23–25°C). One platform was located in the middle of a quadrant submerged 1 cm below the surface of the water. Starting from a semirandom start position, each mouse was tested four times a day for five consecutive days to find a hidden platform. The maximum trial length was 60 s. Each test was terminated if the mouse climbed onto a hidden platform. If the mouse fails to find the platform within 60 s, it was gently guided to the platform and left on the escape platform for 30 s at the end of each trial, then dried and placed back in their home cage. A 60-s probe test without platform was conducted 24 h after the last acquisition session. The swim distance (cm), swim speed (cm/s), escape latency (s) in initial training, swim path, latency to enter the target (platform location area), and the number of platform site zone crossings were recorded through an automated tracking system (Smart video tracking system, Panlab; Harvard Apparatus).

### Immunofluorescence

After behavioral test for memory ability, mice were anesthetized deeply with inhaled isoflurane (1%) and then transcardially perfused with 400 ml 0.9% NaCl followed by 400 ml 4% paraformaldehyde. Then, the brain was removed and postfixed at 4°C in 4% paraformaldehyde for 24 h. Coronal serial sections were cut at 30 mm thickness with a vibratome (VT1000S, Leica, Nussloch, Germany). Free-floating coronal brain sections were washed with 0.01 M PBS (15 min, three times each) and then permeabilized with 1% Triton for 30 min. After washing three times for 10 min with PBS, the brain sections were blocked with 3% bovine serum albumin (BSA) for 60 min. Primary mouse antibody Aβ incubation was performed in blocking solution overnight at 4°C. The sections were again washed in 0.01 M PBS (15 min, three times each) and were incubated with Alexa Fluor 488-conjugated goat antirabbit IgG secondary antibodies (1:100; Abcam) in 0.01 M PBS with 0.05% Tween 20 at room temperature for 2 h. After being washed three times in 0.01 M PBS for 15 min, sections were mounted and incubated with ProLong Gold antifade reagent included 4′,6-diamidino-2-phenylindole (DAPI; CST), then were coverslipped. Imaging was performed on a laser scanning confocal microscope (A1si, Nikon, Japan). All images were analyzed using ImageJ software (NIH, Bethesda, USA).

### Western Blot

After the mice were deeply anesthetized and decapitated; both brain hemispheres were collected and stored in −80°C. The frozen hippocampus and cortex of mice brain were homogenized in prechilled radioimmunoprecipitation assay (RIPA) buffer. The protein concentration of the samples was determined with the BCA kit (Pierce, MA, USA). The same amounts (20–30 μg) of brain protein were separated by sodium dodecyl sulfate–polyacrylamide gel electrophoresis (SDS-PAGE) on 8, 10, or 12% gels and then transferred on to a polyvinylidene difluoride membranes (Roche, Switzerland). The membranes were incubated overnight at 4°C with different primary antibodies, which are list in Table 1. We then incubated the membranes with secondary antibody (1:3,000, Cell Signaling Technology, Danvers, MA, USA) conjugated to horseradish peroxidase (HRP) for 1 h. Glyceraldehyde 3-phosphate dehydrogenase (GAPDH) and b-tubulin levels were analyzed as a loading control. The immunocomplexes were visualized using the Pierce ECL Western blot substrate (Thermo Scientific, Waltham, MA, USA). We visualized the protein bands with automatic chemiluminescence apparatus (BIO-RAD, USA). The specific molecular band intensity was determined using ImageJ.

### Statistical Analysis

**Figure 2:**
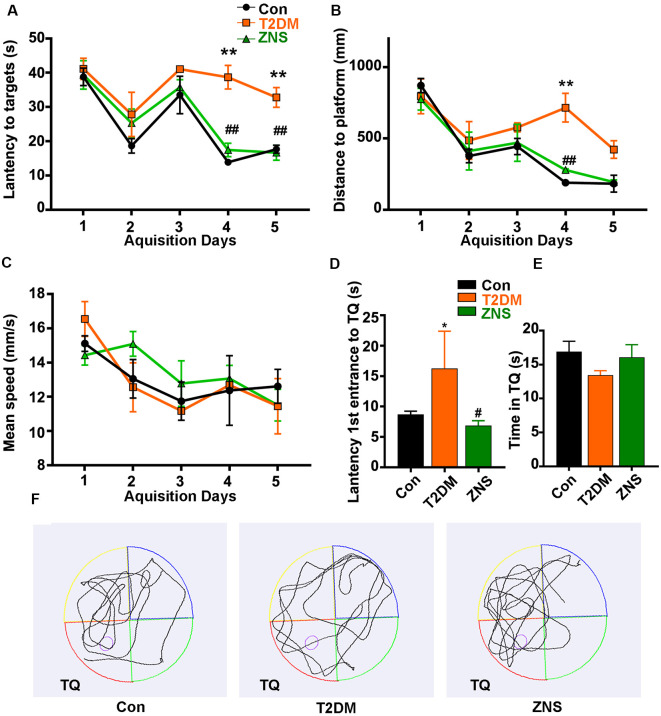
Zonisamide improves learning and memory ability of HFD/STZ-induced mice using Morris water maze (MWM). The effects of Zonisamide on spatial learning in type 2 diabetic mice were evaluated using the MWM for 5 consecutive days. **(A,C)** The travel distance and escape latency during the training phase (D1–D5). Compared to the Con group, the T2DM mice exhibited a significant increase in distance and latency in searching the target platform, which was ameliorated by Zonisamide treatment. **(B)** Mean swim speed. The probe trial was performed on the D6. **(D)** The first latency to reach the platform location. Compared to Con mice, the diabetic mice spent significantly more time to reach the target quadrant, while Zonisamide treatment rescued this memory deficit in diabetic mice. **(E)** The time spent in the target quadrant. **(F)** Representative swimming path tracing of searching for the target quadrant on the fifth day (Tg, target quadrant). Data are presented as mean ± SEM (*n* = 10 mice). *P < 0.05, **P < 0.01 vs. Con and ^#^*P* < 0.05, ^##^*P* < 0.01 vs. T2DM.

Data were analyzed using GraphPad Prism 8.0 (GraphPad Software, Inc., La Jolla, CA, USA). The density values were expressed as the mean ± SEM. Statistical significance was assessed by two-way ANOVA with posttest (Turkey) for data of body glucose, body weight (Figures 1B,C,E), behavior changes in latency and distance (Figures 2A–C), and by one-way ANOVA with posttest (Turkey) for the other data. *P* < 0.05 were considered statistically significant.

## Results

### Zonisamide Ameliorates the Hyperglycemia in T2DM Mice

From the third week after normal or HFD, the body weight and fasting blood glucose of the mice were tested every 2 weeks. A marked increase in fasting blood glucose level was observed in T2DM mice, a slight decrease in the 13th week, and an obvious decrease in the 25th week after Zonisamide treatment (Figure 1B). Besides, we examined the effect of Zonisamide on glucose metabolism with oral glucose tolerance test (OGTT). The data showed a significant increase in glucose concentration in T2DM mice at all time points, which depicted an impaired glucose tolerance, while the glucose levels both at 90 and 120 min declined by Zonisamide treatment (Figure 1C). Furthermore, the higher value of area under the concentration curve (AUC) showed a poor hypoglycemic effect, and we found that the AUC of T2DM mice was significantly increased compared to the control, while Zonisamide treatment also slightly affected the area value (Figure 1D). Meanwhile, from week 3 to 25, the body weight of T2DM mice did not change much during the whole treatment course, while the body weight of Con group mice significantly increased. The body weight of T2DM mice gradually improved after treatment with Zonisamide from the ninth week (Figure 1E).

### Zonisamide Improves Cognitive Impairment in T2DM Mice

The effects of Zonisamide on learning and memory of T2DM mice were assessed using the MWM. In the acquisition trials, T2DM mice spent more distance to reach the target quadrant and platform than the Con mice on the fourth day and fifth day, while compared with the T2DM mice groups, T2DM mice treated with Zonisamide showed a shorter escape latency on days 4–5 (Figures 2A,B). These results demonstrated that Zonisamide ameliorated the deficit of spatial learning in T2DM mice. Furthermore, mean speed was an important index to experimental and control animals, and we also recorded the mean speed of the mice in the sailing test, but no significant difference was observed among all groups (Figure 2C). Then, we performed a probe test to detect the spatial memory of the mice 24 h after the learning ability test. Zonisamide-treated T2DM mice took less time to cross the target quadrant for the first time compared to the vehicle-treated T2DM group mice (Figures 2D,F). The time spent in the target quadrant of the Zonisamide-treated mice was slightly longer than that of the vehicle-treated T2DM mice (Figures 2E,F). These results showed that the Zonisimade-treated T2DM mice had a better memory than the vehicle-treated T2DM mice. Overall, these results indicated that Zonisamide treatment could improve spatial learning and memory in T2DM mice.

### Zonisamide Increases Synaptic-related Proteins Expression in T2DM Mice

**Figure 3:**
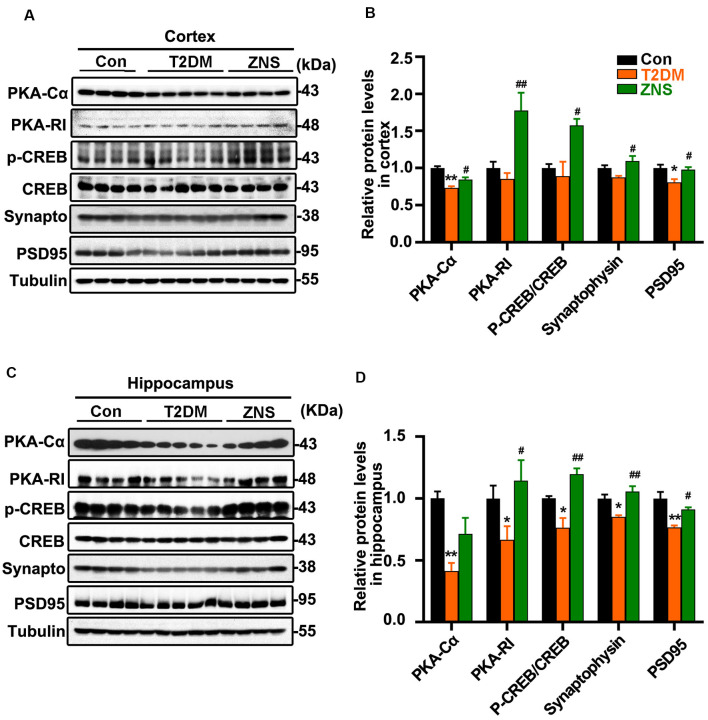
Zonisamide enhances cAMP-dependent protein kinase (PKA)/cyclic AMP response element-binding (CREB) activity and expression of postsynaptic density 95 (PSD95) and syp in the HFD/STZ-induced mice brain. Western blot analyses of synaptophysin (synapto), PSD95, pCREB (pSer133)/CREB, PKA RI, and PKAcα in the hippocampus and cortex. Zonisamide treatment ameliorated deficits in synaptophysin, PSD95, PKA RI, and PKAcα expression, and increased pCREB/CREB ratios. **(A,C)** Representative Western blot. **(B,D)** Quantification of the Western blot. The data are presented as mean ± SEM (*n* = 4–5 mice). *P < 0.05, **P < 0.01 vs. Con; ^#^*P* < 0.05, ^##^*P* < 0.01 vs. T2DM.

Synapses are the structural basis of memory and cognition. Postsynaptic density 95 (PSD95) is implicated in experience-dependent plasticity and plays an indispensable role in learning (Yao et al., 2004). In order to evaluate whether Zonisamide treatment influenced the synapse, we tested the expressions of synaptophysin and the postsynaptic protein (PSD95) for possible synaptic changes (Figures 3A,C). The levels of PSD95 were decreased both in the cortex and hippocampus of vehicle-treated T2DM mice compared to the control group mice, while synaptophysin was also reduced in the hippocampus and there was a trend in the decrease in synaptophysin in the cortex. Moreover, Zonisamide significantly increased the expression of PSD95 and synaptophysin both in the hippocampus and cortex of T2DM mice (Figures 3B,D).

PKA–cyclic amp response element-binding (CREB) signaling plays an important role in learning and long-term memory, and the CREB activity is mainly regulated by its phosphorylated at Ser 133. We investigated whether PKA/CREB signaling was affected in T2DM mice with Zonisamide treatment (Figures 3A,C). We found that the levels of PKA-RI, PKA-Cα, and phosphorylated CREB were significantly increased in both the cortex and hippocampus of Zonisamide-treated mice (Figures 3B,D). Although the increase in PKA-Cα in the hippocampus is not statistically significant, the trend is clear. These data support that cognition improvement is associated with the PKA–CREB signaling.

### Zonisamide Attenuates Aβ Formation and Tau Phosphorylation in T2DM Mice

**Figure 4:**
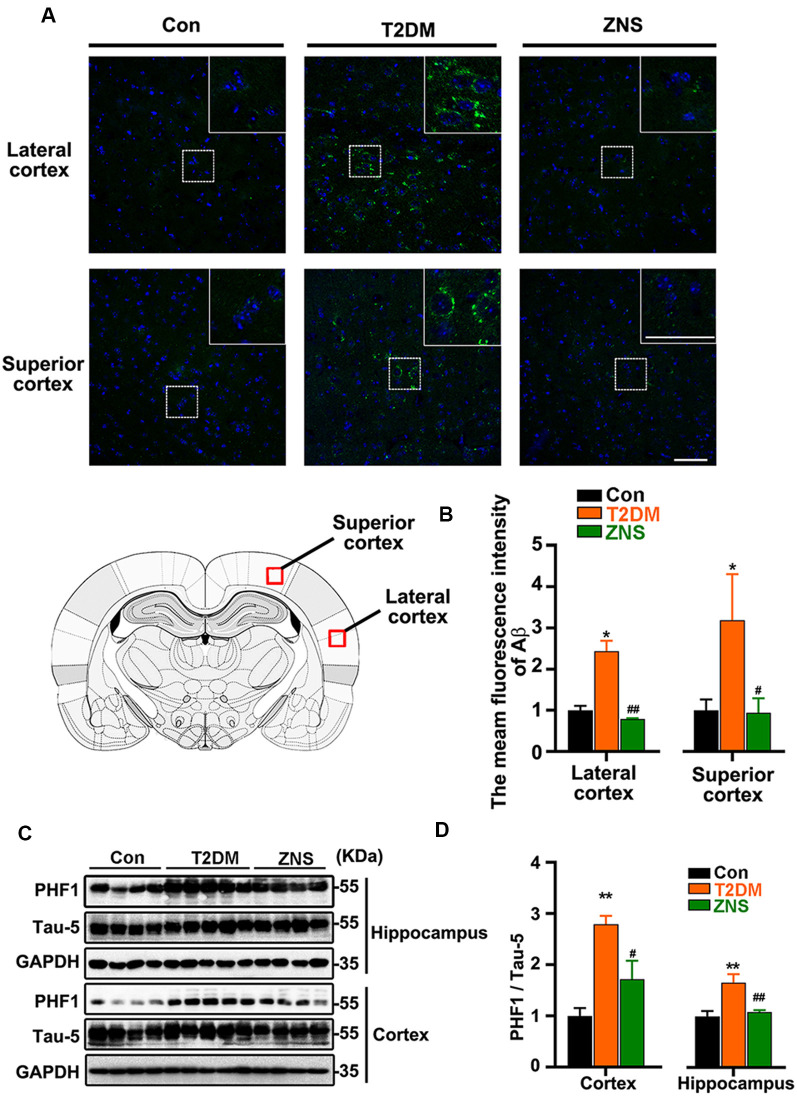
Zonisamide attenuates the accumulation of Aβ and the phosphorylation of Tau in HFD/STZ-induced type 2 diabetic mice. Aβ was detected by Immunofluorescence in the external pyramidal layer (layer III) of the cerebral cortex. PHF1 and total Tau expression in the hippocampus and cortex were analyzed using Western blot. PHF1/Tau-5 expression was increased both in the hippocampus and cortex of T2DM mice, and the staining of Aβ was increased both in the superior and lateral cortex, while Zonisamide treatment attenuated Tau and Aβ alterations. **(A)** Immunofluorescent staining of Aβ (Green) and 4′,6-diamidino-2-phenylindole (DAPI; blue) in the superior and lateral cortex (scale bar = 50 μm). **(B)** Mean fluorescence intensity analysis for Aβ (*n* = 3 per group). **(C)** PHF1 and Tau-5 expression in the cortex and hippocampus. **(D)** The quantitative analysis of PHF1 using Tau-5 as normalization, respectively. Data are presented as the mean ± SEM (*n* = 4–5 mice). *P < 0.05, **P < 0.01 vs. Con; ^#^*P* < 0.05, ^##^*P* < 0.01 vs. T2DM.

To examine the effect of Zonisamide on amyloid β (Aβ) in the superior and lateral cortex, we performed immunofluorescence (Figures 4A,B). We found that Aβ-positive spots were located mainly in the external pyramidal layer of superior cortex and lateral cortex in vehicle-treated T2DM group, and the positive puncta staining were distributed in the cytoplasm and extracellular area. No positive puncta staining formation was observed in the control mice. Treatment of Zonisamide significantly decreased the fluorescence intensity of Aβ in T2DM mice. We also assessed the phosphorylated Tau levels in the cortex and hippocampus of T2DM mice by Western blot (Figure 4C). As the phosphorylation of Tau at Ser 396 and Ser 404 was a critical hallmark in diabetic models (Yang et al., 2013a,b), the site-specific phosphorylated Tau (pSer396/404) was examined by using PHF1 antibodies. PHF1 level was increased obviously in the vehicle-treated T2DM group compared with those of the control group. While in contrast to vehicle-treated T2DM group, PHF1 was decreased with Zonisamide treatment (Figure 4D), which suggests that Tau phosphorylation at Ser 396/404 was significantly decreased by Zonisamide treatment.

**Figure 5:**
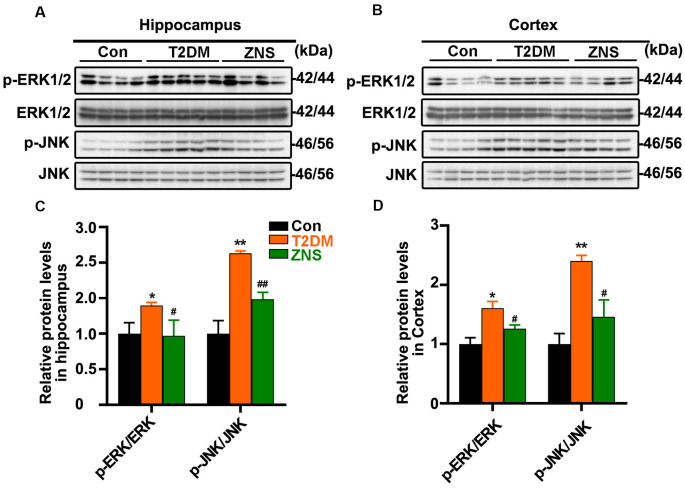
Impact of Zonisamide on extracellular signal-regulated kinase (ERK) 1/2 and c-Jun N-terminal kinase (JNK) in HFD/STZ-induced type 2 diabetic mice. The Tau kinases were assessed by expression of p-ERK1/2 and p-JNK. **(A,B)** The relative levels of p-ERK1/2, p-JNK, ERK1/2, and JNK in the hippocampus and cortex were detected by Western blot. **(C,D)** The quantitative analysis of p-ERK1/2 and p-JNK using ERK1/2 and JNK as normalization, respectively. Data are presented as the means ± SEM (*n* = 4–5 mice). *P < 0.05, **P < 0.01 vs. Con; ^#^*P* < 0.05, ^##^*P* < 0.01 vs. T2DM.

In addition, to further clarify the effect of Zonisamide on upstream kinases of Tau, we examined the protein kinases commonly involved in the regulation of specific Tau phosphorylation at Ser 396/404. We investigate levels of p-ERK1/2 and p-Jun N-terminal kinase (JNK) as well as their total protein (Figures 5A,C). The levels of p-ERK1/2 and p-JNK were both increased, and p-GSK3b (Ser9), which is the inactivated site of GSK-3b, was decreased in T2DM group, while p-ERK1/2 and p-JNK were decreased after Zonisamide treatment. The results suggest that activities of extracellular signal-regulated kinase (ERK) and JNK were significantly increased in T2DM mice compared with the control group, but their activities reversed back to the normal level similar to those in control group mice after Zonisamide treatment (Figures 5B,D).

These results demonstrated that STZ and HFD treatment resulted in Tau hyperphosphorylation in the mice, while Zonisamide might ameliorate the hyperphosphorylation of Tau through regulating the activities of ERK and JNK.

### Zonisamide Increases Hrd1 Levels and Suppresses the ERS in the Brain of T2DM Mice

**Figure 6:**
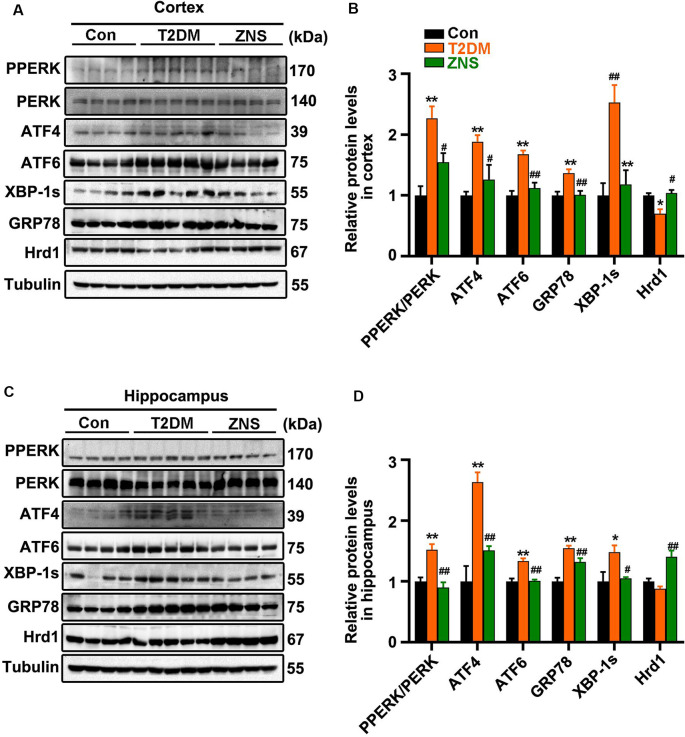
Zonisamide increases Hrd1 levels and suppresses the endoplasmic reticulum stress (ERS) in the brain of type 2 diabetic mice. **(A,C)** Representative and quantitative images of Hrd1, and ERS markers, including GRP78, XBP-1s, ATF6, p-PERK, PERK, and ATF4. **(B,D)** Quantification of the Western blot. All values were the fold changes by normalized to their control group. Data are presented as the mean ± SEM (*n* = 4–5 mice). *P < 0.05, **P < 0.01 vs. Con; ^#^*P* < 0.05, ^##^*P* < 0.01 vs. T2DM.

To ascertain whether excessive ERS is triggered in the brain of type 2 diabetic mice, the relative abundance of several ER stress protein markers [XBP-1s, PPERK, PERK, glucose-regulated protein 78 (GRP78), ATF4, and ATF6] was analyzed by Western blot (Figures 6A,C). The level of XBP-1s, GRP78, ATF4, and ATF6, as well as the phosphorylation level of PERK were significantly increased in vehicle-treated T2DM mice, while the levels of the above proteins were significantly reduced in Zonisamide-treated T2DM mice (Figures 6B,D). Furthermore, we considered the possibility that Zonisamide might suppress ER stress by upregulating the Hrd1 protein levels. Noticeably, hrd1 levels were evidently reduced in the cortex of T2DM mice but did not change significantly just with a trend in decrease in the hippocampus, while Hrd1 was upregulated both in the cortex and hippocampus in Zonisamide-treated mice (Figures 6B,D). Together, these data strongly suggest that Hrd1 and ER stress were involved in the brain of Zonisamide-treated mice.

## Discussion

There is sufficient evidence that type 2 diabetes increases the risk of multi-infarct dementia, AD, and mixed dementia (Kravitz et al., 2013). There is currently no available effective drug for mild cognitive impairment and AD. In addition, whether antidiabetic agent treatment (Bloemer et al., 2014) or reducing glucose level (Cukierman-Yaffe et al., 2009) is helpful in reducing diabetes-related cognitive impairment is not clear. Therefore, the effective drugs to prevent cognitive impairment in type 2 diabetes patients are important.

Common findings of T2DM are hyperglycemia and insulin resistance, and multiple studies have shown increased risk of Alzheimer’s and vascular dementia in patients with impaired glucose tolerance (Kuusisto et al., 1997; Curb et al., 1999). In this study, the fasting blood glucose level and oral glucose tolerance of T2DM group mice were improved after treating with Zonisamide for 16 weeks, although the blood glucose could not fall to normal levels in Zonisamide treatment mice.

Several studies have shown subtle cognitive changes in different cognitive domains in patients with T2DM, particularly in the domains of processing speed, executive function, working memory, immediate recall, delayed recall, verbal, and visual memory (Gregg et al., 2000; Kanaya et al., 2004; van den Berg et al., 2010; Palta et al., 2014). The spatial reference memory task (MWM test) is known to test memory storage, consolidation, and restitution of the spatial information (Moser and Moser, 1998). In this study, we observed that Zonisamide could improve the learning and memory ability in the acquisition trials and probe testing, as evidenced by shorter escape latencies, shorter distance to reach the platform, and less time to cross the target quadrant in Zonisamide-treated mice. These results suggest that Zonisamide treatment enhanced memory consolidation in T2DM mice.

The phosphorylation of CREB plays an important role in synaptic plasticity and long-term memory (Bailey et al., 1996). In addition, STZ has been indicated as selective postsynaptic neurotoxicity (Rai et al., 2014). Consistent with the previous study (Arnold et al., 2014; Rai et al., 2014; Carvalho et al., 2015; Zhao et al., 2018), we observed the decreased expression of p-CREB, PKA-cα, synaptophysin, and PSD95 in type 2 diabetes mice. These deficits were rescued by the administration of Zonisamide, hinting that Zonisamide might have ameliorated learning and memory deficits in T2DM mice by one of these pathways.

It has been reported that animal models of diabetes exhibit accumulation of Aβ (Li et al., 2007; Mehla et al., 2014; Kang et al., 2017). We also found that Aβ deposition was observed in the cortex of the T2DM mice, while the Aβ load was obviously attenuated after Zonisamide treatment. Previous studies showed that JNK pathway was activated by ER stress in animal models of obese (Liang et al., 2015) and T2DM (Sims-Robinson et al., 2012), resulting in impaired insulin receptor signaling in the hippocampus and cortex, exhibiting an increased activity of GSK3β and Tau hyperphosphorylation (Chiu and Cline, 2010; Barbagallo and Dominguez, 2014; Ma et al., 2014). The sites at Ser 396/404 of Tau are very important, and phosphorylated Tau at Ser 396/404 are increased both in the cortex and hippocampus in T2DM (Kim et al., 2009; Yang et al., 2013a,b). Moreover, phosphorylation of Tau protein at sites Ser(396/404) is an efficient marker for AD cytopathology (Mondragón-Rodríguez et al., 2014). Thus, we investigated the influence of Zonisamide on Tau phosphorylation at 396/404 sites, which has been described as an important contributor to early phospho-Tau aggregates. In this study, consistent with previous findings (Kim et al., 2009; Kim and Feldman, 2012), we found that expression levels of p-Tau at Ser 396/404 were remarkably increased both in the hippocampus and cortex, while Zonisamide treatment attenuated Tau phosphorylation in T2DM mice. Furthermore, it was identified that the site-specific phosphorylation (Ser396/404) of Tau was activated by ERK1/2 and JNK (Reynolds et al., 2000; Yarza et al., 2016). Results showed that p-ERK and p-JNK were increased significantly, which means that the ERK and JNK were activated. Meanwhile, Zonisamide reduced the activities of these Tau kinases in T2DM mice. In brief, there is a possibility that Zonisamide may exert its disease-modifying effect on Tau hyperphosphorylation by mainly decreasing the activity of JNK.

Previous evidence supported that the ER-stress-induced unfolded protein responses (UPRs) are essential steps in diabetes mediated neurotoxicity and cognitive impairment (Lind et al., 2013; Zhang et al., 2013; Zhao et al., 2015; Kong et al., 2018); the ER stress markers (ATF4, GRP78, ATF6, P-PERK, XBP-1s) are all increased both in the hippocampus and cortex in the animal model of diabetes. Evidence suggests that PERK is directly responsible for cognitive impairment in AD patients (Duran-Aniotz et al., 2014), and both the activation of PERK (Lin et al., 2018) and ATF4 expression (Chen et al., 2003) have been shown to impair the transcriptional activity of CREB, leading to synaptic plasticity inhibition. In addition, it was identified that phosphorylation of PSD95 induced by PERK leads to degradation of PSD95 (Sen et al., 2017). Recently, it has been demonstrated that Zonisamide inhibited neuronal cell death caused by ER stress (Omura et al., 2012; Tsujii et al., 2015). In the study, the ER stress markers (ATF4, GRP78, ATF6, P-PERK, XBP-1s) were significantly increased in T2DM mice, and these effects were abolished by the administration of Zonisamide. In addition, we observed a significant upregulation of Hrd1 expression in Zonisamide treatment mice. Hrd1 (an E3 ubiquitin ligase), an important component of ERAD, plays a very broad role in the ubiquitination process of abnormal proteins and is involved in clearing irreparably misfolded proteins activated by UPR (Omura et al., 2008), and protein levels of HRD1 are upregulated in response to ER stress. However, the Hrd1 protein expression under the ER stress in the brain remains unclear. It has been reported that the protein levels of Hrd1 in the cortex are decreased both in diabetic rats and in AD brain compared to the control group rat and non-AD subjects, and the brains of these diabetic rats and AD patients experienced ER stress (Kaneko et al., 2010; Shruthi et al., 2019). ER is involved in the processing and trafficking of amyloid precursor protein (APP); once in the ER, APP can generate Aβ due to the γ-secretases (Plácido et al., 2014). In addition, the breakdown of Hrd1-mediated reticulum-associated degradation (ERAD) causes Aβ generation and ER stress (Kaneko et al., 2010). In addition, Hrd1 interact with APP through the proline-rich region as well as promote APP ubiquitination and degradation, resulting in the decreased generation of Aβ. Thus, ERS and Hrd1 are involved in the regulation of the APP and reduction in Aβ (Kaneko et al., 2010, 2012). Thus, Zonisamide may have promoted Hrd1-mediated ERAD through the upregulation of Hrd1, resulting in the improvement of ER stress and decreased the generation of Aβ.

Consistent with the previous study, we also found that the expression levels of Hrd1 in cortex was reduced in T2DM mice. Previous study revealed that this decrease in Hrd1 was due to its insolubilization, and it remains unclear how Hrd1 protein is insolubilized (Kaneko et al., 2012). In addition, normal levels of Hrd1 are also observed in some non-AD patients with high levels of Aβ (Saito et al., 2010). Thus, no alterations of Hrd1 were observed in the hippocampus of T2DM mice, probably because the change in hrd1 is still in the compensation period. Interestingly, Zonisamide increased Hrd1 level significantly both in the hippocampus and cortex and decreased the Aβ load in cortex of T2DM mice. Thus, it is possible that Zonisamide normalized the ER stress and Aβ by virtue of its upregulation effect on Hrd1 in T2DM mice. The upregulation of Hrd1 might be caused by SEL1L activation (Omura et al., 2013).

## Conclusion

In conclusion, the present study indicates for the first time that Zonisamide may ameliorate the cognitive impairment through increasing the PSD95 and CREB in T2DM mice, decrease the Aβ load, and rescue the Tau hyperphosphorylation at Ser 396/404 through inhibiting the activity of JNK. The above results may be related to the regulation of ER stress through the elevation of Hrd1 by Zonisamide. Therefore, Zonisamide may become a new reagent for the treatment of dementia in T2DM.

## Data Availability Statement

The original contributions presented in the study are included in the article, further inquiries can be directed to the corresponding author/s.

## Ethics Statement

The animal study was reviewed and approved by Committee on the Ethics of Animal Experiments of Guangzhou Medical University and the approval reference number was SYXK 2016-0168.

## Author Contributions

Y-XH carried out the study and wrote the manuscript. Q-YS performed part of the experiments and wrote the article. J-HT treated the animals and analyzed the data. QW and G-PZ treated the animals and participated in the animal experiment. QX helped in discussing the data and writing the manuscript. Y-HL and WW conceived, designed, and supervised the study and wrote the article.

## Conflict of Interest

The authors declare that the research was conducted in the absence of any commercial or financial relationships that could be construed as a potential conflict of interest.

## Abbreviations

Aβ: amyloid β
AD: Alzheimer’s disease
ATF4: activating transcription factor-4
ATF6: activating transcription factor-6
CREB: cyclic AMP response element-binding
ERK1/2: extracellular regulated protein kinases
ERS: endoplasmic reticulum stress
GRP78: glucose-regulated protein 78
GSK3β: glycogen synthase kinase-3
HFD: high-fat diet
Hrd1: HMG-CoA reductase degradation 1
JNK: c-Jun N-terminal kinase
PERK: protein kinase R-like ER kinase
PSD95: postsynaptic protein
PKA: cAMP-dependent protein kinase
STZ: streptozotocin
T2DM: type 2 diabetes mellitus
URP: unfolded protein response
XBP-1s: X-Box binding protein-1s
ZNS: Zonisamide.

## References

[B1] Arnold S. E. Lucki I. Brookshire B. R. Carlson G. C. Browne C. A. Kazi H.. (2014). High fat diet produces brain insulin resistance, synaptodendritic abnormalities and altered behavior in mice. Neurobiol. Dis. 67, 79–87. 10.1016/j.nbd.2014.03.011 24686304 PMC4083060

[B2] Bailey C. H. Bartsch D. Kandel E. R. (1996). Toward a molecular definition of long-term memory storage. Proc. Natl. Acad. Sci. U S A 93, 13445–13452. 10.1073/pnas.93.24.13445 8942955 PMC33629

[B3] Barbagallo M. Dominguez L. J. (2014). Type 2 diabetes mellitus and Alzheimer’s disease. World J. Diabetes 5, 889–893. 10.4239/wjd.v5.i6.889 25512792 PMC4265876

[B4] Bitel C. L. Kasinathan C. Kaswala R. H. Klein W. L. Frederikse P. H. (2012). Amyloid-β and tau pathology of Alzheimer’s disease induced by diabetes in a rabbit animal model. J. Alzheimers Dis. 32, 291–305. 10.3233/JAD-2012-120571 22785400

[B5] Bloemer J. Bhattacharya S. Amin R. Suppiramaniam V. (2014). Impaired insulin signaling and mechanisms of memory loss. Prog. Mol. Biol. Transl. Sci. 121, 413–449. 10.1016/b978-0-12-800101-1.00013-2 24373245

[B6] Carvalho C. Santos M. S. Oliveira C. R. Moreira P. I. (2015). Alzheimer’s disease and type 2 diabetes-related alterations in brain mitochondria, autophagy and synaptic markers. Biochim. Biophys. Acta 1852, 1665–1675. 10.1016/j.bbadis.2015.05.001 25960150

[B7] Chen A. Muzzio I. A. Malleret G. Bartsch D. Verbitsky M. Pavlidis P.. (2003). Inducible enhancement of memory storage and synaptic plasticity in transgenic mice expressing an inhibitor of ATF4 (CREB-2) and C/EBP proteins. Neuron 39, 655–669. 10.1016/s0896-6273(03)00501-4 12925279

[B8] Chiu S.-L. Cline H. T. (2010). Insulin receptor signaling in the development of neuronal structure and function. Neural Dev. 5:7. 10.1186/1749-8104-5-7 20230616 PMC2843688

[B9] Cukierman-Yaffe T. Gerstein H. C. Williamson J. D. Lazar R. M. Lovato L. Miller M. E.. (2009). Relationship between baseline glycemic control and cognitive function in individuals with type 2 diabetes and other cardiovascular risk factors: the action to control cardiovascular risk in diabetes-memory in diabetes (ACCORD-MIND) trial. Diabetes Care 32, 221–226. 10.2337/dc08-1153 19171735 PMC2628683

[B10] Curb J. D. Rodriguez B. L. Abbott R. D. Petrovitch H. Ross G. W. Masaki K. H.. (1999). Longitudinal association of vascular and Alzheimer’s dementias, diabetes, and glucose tolerance. Neurology 52, 971–975. 10.1212/wnl.52.5.971 10102414

[B11] Duran-Aniotz C. Martinez G. Hetz C. (2014). Memory loss in Alzheimer’s disease: are the alterations in the UPR network involved in the cognitive impairment? Front. Aging Neurosci. 6:8. 10.3389/fnagi.2014.00008 24523695 PMC3906588

[B12] Feinkohl I. Price J. F. Strachan M. W. Frier B. M. (2015). The impact of diabetes on cognitive decline: potential vascular, metabolic, and psychosocial risk factors. Alzheimers Res. Ther. 7:46. 10.1186/s13195-015-0130-5 26060511 PMC4460635

[B13] Greenberg S. G. Davies P. Schein J. D. Binder L. I. (1992). Hydrofluoric acid-treated tau PHF proteins display the same biochemical properties as normal tau. J. Biol. Chem. 267, 564–569. 1370450

[B14] Gregg E. W. Yaffe K. Cauley J. A. Rolka D. B. Blackwell T. L. Narayan K. M.. (2000). Is diabetes associated with cognitive impairment and cognitive decline among older women? Study of Osteoporotic Fractures Research Group. Arch. Intern. Med. 160, 174–180. 10.1001/archinte.160.2.174 10647755

[B15] Hetz C. Chevet E. Harding H. P. (2013). Targeting the unfolded protein response in disease. Nat. Rev. Drug Discov. 12, 703–719. 10.1038/nrd3976 23989796

[B16] Holder J. L. Jr. Wilfong A. A. (2011). Zonisamide in the treatment of epilepsy. Expert Opin. Pharmacother. 12, 2573–2581. 10.1517/14656566.2011.622268 21967409

[B17] Kanaya A. M. Barrett-Connor E. Gildengorin G. Yaffe K. (2004). Change in cognitive function by glucose tolerance status in older adults: a 4-year prospective study of the Rancho Bernardo study cohort. Arch. Intern. Med. 164, 1327–1333. 10.1001/archinte.164.12.1327 15226167

[B18] Kandimalla R. Thirumala V. Reddy P. H. (2017). Is Alzheimer’s disease a Type 3 Diabetes? A critical appraisal. Biochim. Biophys. Acta Mol. Basis Dis. 1863, 1078–1089. 10.1016/j.bbadis.2016.08.018 27567931 PMC5344773

[B19] Kaneko M. Koike H. Saito R. Kitamura Y. Okuma Y. Nomura Y. (2010). Loss of HRD1-mediated protein degradation causes amyloid precursor protein accumulation and amyloid-β generation. J. Neurosci. 30, 3924–3932. 10.1523/jneurosci.2422-09.2010 20237263 PMC6632277

[B20] Kaneko M. Okuma Y. Nomura Y. (2012). Molecular approaches to the treatment, prophylaxis, and diagnosis of Alzheimer’s disease: possible involvement of HRD1, a novel molecule related to endoplasmic reticulum stress, in Alzheimer’s disease. J. Pharmacol. Sci. 118, 325–330. 10.1254/jphs.11r11fm 22382662

[B22] Kang S. Kim C.-H. Jung H. Kim E. Song H.-T. Lee J. E. (2017). Agmatine ameliorates type 2 diabetes induced-Alzheimer’s disease-like alterations in high-fat diet-fed mice *via* reactivation of blunted insulin signalling. Neuropharmacology 113, 467–479. 10.1016/j.neuropharm.2016.10.029 27810390

[B24] Kim B. Backus C. Oh S. Hayes J. M. Feldman E. L. (2009). Increased tau phosphorylation and cleavage in mouse models of type 1 and type 2 diabetes. Endocrinology 150, 5294–5301. 10.1210/en.2009-0695 19819959 PMC2795717

[B23] Kim B. Feldman E. L. (2012). Insulin resistance in the nervous system. Trends Endocrinol. Metab. 23, 133–141. 10.1016/j.tem.2011.12.004 22245457 PMC3392648

[B25] Kong F.-J. Ma L.-L. Guo J.-J. Xu L.-H. Li Y. Qu S. (2018). Endoplasmic reticulum stress/autophagy pathway is involved in diabetes-induced neuronal apoptosis and cognitive decline in mice. Clin. Sci. 132, 111–125. 10.1042/cs20171432 29212786

[B26] Kravitz E. Schmeidler J. Schnaider Beeri M. (2013). Type 2 diabetes and cognitive compromise: potential roles of diabetes-related therapies. Endocrinol. Metab. Clin. North Am. 42, 489–501. 10.1016/j.ecl.2013.05.009 24011882 PMC3767929

[B27] Kuusisto J. Koivisto K. Mykkänen L. Helkala E. L. Vanhanen M. Hanninen T.. (1997). Association between features of the insulin resistance syndrome and Alzheimer’s disease independently of apolipoprotein E4 phenotype: cross sectional population based study. BMJ 315, 1045–1049. 10.1136/bmj.315.7115.1045 9366728 PMC2127678

[B28] Lai E. Teodoro T. Volchuk A. (2007). Endoplasmic reticulum stress: signaling the unfolded protein response. Physiology 22, 193–201. 10.1152/physiol.00050.2006 17557940

[B29] Li Z. G. Zhang W. Sima A. A. (2007). Alzheimer-like changes in rat models of spontaneous diabetes. Diabetes 56, 1817–1824. 10.2337/db07-0171 17456849

[B30] Liang L. Chen J. Zhan L. Lu X. Sun X. Sui H.. (2015). Endoplasmic reticulum stress impairs insulin receptor signaling in the brains of obese rats. PLoS One 10:e0126384. 10.1371/journal.pone.0126384 25978724 PMC4433117

[B31] Lin L. Cao J. Yang S. S. Fu Z. Q. Zeng P. Chu J.. (2018). Endoplasmic reticulum stress induces spatial memory deficits by activating GSK-3. J. Cell. Mol. Med. 22, 3489–3502. 10.1111/jcmm.13626 29675957 PMC6010738

[B32] Lind K. R. Ball K. K. Cruz N. F. Dienel G. A. (2013). The unfolded protein response to endoplasmic reticulum stress in cultured astrocytes and rat brain during experimental diabetes. Neurochem. Int. 62, 784–795. 10.1016/j.neuint.2013.02.009 23411409 PMC3615116

[B33] Ma L. Zhao Z. Wang R. Zhang X. Zhang J. Dong W.. (2014). Caloric restriction can improve learning ability in C57/BL mice *via* regulation of the insulin-PI3K/Akt signaling pathway. Neurol. Sci. 35, 1381–1386. 10.1007/s10072-014-1717-5 24651932

[B34] Mehla J. Chauhan B. C. Chauhan N. B. (2014). Experimental induction of type 2 diabetes in aging-accelerated mice triggered Alzheimer-like pathology and memory deficits. J. Alzheimers Dis. 39, 145–162. 10.3233/jad-131238 24121970 PMC3941701

[B35] Mondragón-Rodríguez S. Perry G. Luna-Muñoz J. Acevedo-Aquino M. C. Williams S. (2014). Phosphorylation of tau protein at sites Ser(396–404) is one of the earliest events in Alzheimer’s disease and Down syndrome. Neuropathol. Appl. Neurobiol. 40, 121–135. 10.1111/nan.12084 24033439

[B36] Moser M. B. Moser E. I. (1998). Distributed encoding and retrieval of spatial memory in the hippocampus. J. Neurosci. 18, 7535–7542. 10.1523/JNEUROSCI.18-18-07535.1998 9736671 PMC6793256

[B37] Omura T. Asari M. Yamamoto J. Kamiyama N. Oka K. Hoshina C.. (2012). HRD1 levels increased by zonisamide prevented cell death and caspase-3 activation caused by endoplasmic reticulum stress in SH-SY5Y cells. J. Mol. Neurosci. 46, 527–535. 10.1007/s12031-011-9638-8 21892618

[B38] Omura T. Kaneko M. Okuma Y. Matsubara K. Nomura Y. (2013). Endoplasmic reticulum stress and Parkinson’s disease: the role of HRD1 in averting apoptosis in neurodegenerative disease. Oxid. Med. Cell. Longev. 2013:239854. 10.1155/2013/239854 23710284 PMC3654363

[B39] Omura T. Kaneko M. Tabei N. Okuma Y. Nomura Y. (2008). Immunohistochemical localization of a ubiquitin ligase HRD1 in murine brain. J. Neurosci. Res. 86, 1577–1587. 10.1002/jnr.21616 18241051

[B40] Palta P. Schneider A. L. C. Biessels G. J. Touradji P. Hill-Briggs F. (2014). Magnitude of cognitive dysfunction in adults with type 2 diabetes: a meta-analysis of six cognitive domains and the most frequently reported neuropsychological tests within domains. J. Int. Neuropsychol. Soc. 20, 278–291. 10.1017/s1355617713001483 24555960 PMC4132660

[B41] Plácido A. I. Pereira C. M. Duarte A. I. Candeias E. Correia S. C. Santos R. X.. (2014). The role of endoplasmic reticulum in amyloid precursor protein processing and trafficking: implications for Alzheimer’s disease. Biochim. Biophys. Acta 1842, 1444–1453. 10.1016/j.bbadis.2014.05.003 24832819

[B42] Pugazhenthi S. Qin L. Reddy P. H. (2017). Common neurodegenerative pathways in obesity, diabetes and Alzheimer’s disease. Biochim. Biophys. Acta Mol. Basis Dis. 1863, 1037–1045. 10.1016/j.bbadis.2016.04.017 27156888 PMC5344771

[B43] Rai S. Kamat P. K. Nath C. Shukla R. (2014). Glial activation and post-synaptic neurotoxicity: the key events in Streptozotocin (ICV) induced memory impairment in rats. Pharmacol. Biochem. Behav. 117, 104–117. 10.1016/j.pbb.2013.11.035 24333387

[B45] Reynolds C. H. Betts J. C. Blackstock W. P. Nebreda A. R. Anderton B. H. (2000). Phosphorylation sites on tau identified by nanoelectrospray mass spectrometry. J. Neurochem. 74, 1587–1595. 10.1046/j.1471-4159.2000.0741587.x 10737616

[B46] Saito R. Kaneko M. Okuma Y. Nomura Y. (2010). Correlation between decrease in protein levels of ubiquitin ligase HRD1 and amyloid-β production. J. Pharmacol. Sci. 113, 285–288. 10.1254/jphs.10118sc 20606367

[B47] Seino M. (2004). Review of zonisamide development in Japan. Seizure 13, S2–S4. 10.1016/j.seizure.2004.04.015 15511683

[B48] Sen T. Gupta R. Kaiser H. Sen N. (2017). Activation of PERK elicits memory impairment through inactivation of CREB and downregulation of PSD95 following traumatic brain injury. J. Neurosci. 37, 5900–5911. 10.1523/JNEUROSCI.2343-16.2017 28522733 PMC5473207

[B49] Shruthi K. Reddy S. S. Chitra P. S. Reddy G. B. (2019). Ubiquitin-proteasome system and ER stress in the brain of diabetic rats. J. Cell. Biochem. 120, 5962–5973. 10.1002/jcb.27884 30317658

[B50] Sims-Robinson C. Zhao S. Hur J. Feldman E. L. (2012). Central nervous system endoplasmic reticulum stress in a murine model of type 2 diabetes. Diabetologia 55, 2276–2284. 10.1007/s00125-012-2573-6 22581041 PMC3391332

[B51] Tsujii S. Ishisaka M. Shimazawa M. Hashizume T. Hara H. (2015). Zonisamide suppresses endoplasmic reticulum stress-induced neuronal cell damage *in vitro* and *in vivo*. Eur. J. Pharmacol. 746, 301–307. 10.1016/j.ejphar.2014.09.023 25261037

[B52] Vagelatos N. T. Eslick G. D. (2013). Type 2 diabetes as a risk factor for Alzheimer’s disease: the confounders, interactions, and neuropathology associated with this relationship. Epidemiol. Rev. 35, 152–160. 10.1093/epirev/mxs012 23314404

[B53] van den Berg E. Reijmer Y. D. de Bresser J. Kessels R. P. Kappelle L. J. Biessels G. J. (2010). A 4 year follow-up study of cognitive functioning in patients with type 2 diabetes mellitus. Diabetologia 53, 58–65. 10.1007/s00125-009-1571-9 19882137 PMC2789935

[B54] Yang Y. Ma D. Wang Y. Jiang T. Hu S. Zhang M.. (2013a). Intranasal insulin ameliorates tau hyperphosphorylation in a rat model of type 2 diabetes. J. Alzheimers Dis. 33, 329–338. 10.3233/jad-2012-121294 22936005

[B55] Yang Y. Zhang J. Ma D. Zhang M. Hu S. Shao S.. (2013b). Subcutaneous administration of liraglutide ameliorates Alzheimer-associated tau hyperphosphorylation in rats with type 2 diabetes. J. Alzheimers Dis. 37, 637–648. 10.3233/jad-130491 23948890

[B44] Yarza R. Vela S. Solas M. Ramirez M. J. (2016). c-Jun N-terminal kinase (JNK) signaling as a therapeutic target for Alzheimer’s disease. Front. Pharmacol. 6:321. 10.3389/fphar.2015.00321 26793112 PMC4709475

[B56] Yao W. D. Gainetdinov R. R. Arbuckle M. I. Sotnikova T. D. Cyr M. Beaulieu J. M.. (2004). Identification of PSD-95 as a regulator of dopamine-mediated synaptic and behavioral plasticity. Neuron 41, 625–638. 10.1016/s0896-6273(04)00048-0 14980210

[B57] Zhang X. Xu L. He D. Ling S. (2013). Endoplasmic reticulum stress-mediated hippocampal neuron apoptosis involved in diabetic cognitive impairment. Biomed. Res. Int. 2013:924327. 10.1155/2013/924327 23710464 PMC3655482

[B58] Zhao L. Dong M. Ren M. Li C. Zheng H. Gao H. (2018). Metabolomic analysis identifies lactate as an important pathogenic factor in diabetes-associated cognitive decline rats. Mol. Cell. Proteomics 17:2335. 10.1074/mcp.ra118.000690 30171160 PMC6283288

[B59] Zhao Y. Yan Y. Zhao Z. Li S. Yin J. (2015). The dynamic changes of endoplasmic reticulum stress pathway markers GRP78 and CHOP in the hippocampus of diabetic mice. Brain Res. Bull. 111, 27–35. 10.1016/j.brainresbull.2014.12.006 25529350

